# Proteomics and Metabolomics Studies on the Biotic Stress Responses of Rice: an Update

**DOI:** 10.1186/s12284-021-00461-4

**Published:** 2021-03-15

**Authors:** Kieu Thi Xuan Vo, Md Mizanor Rahman, Md Mustafizur Rahman, Kieu Thi Thuy Trinh, Sun Tae Kim, Jong-Seong Jeon

**Affiliations:** 1 Graduate School of Biotechnology and Crop Biotech Institute, Kyung Hee University, Yongin, 17104 South Korea; 2 Department of Plant Bioscience, Pusan National University, Miryang, 50463 South Korea

**Keywords:** Proteomics, Metabolomics, Biotic stress, *Magnaporthe oryzae*, Brown Plant hopper, *Xanthomonas oryzae*, Rice stripe virus, Nematode, Rice

## Abstract

Biotic stresses represent a serious threat to rice production to meet global food demand and thus pose a major challenge for scientists, who need to understand the intricate defense mechanisms. Proteomics and metabolomics studies have found global changes in proteins and metabolites during defense responses of rice exposed to biotic stressors, and also reported the production of specific secondary metabolites (SMs) in some cultivars that may vary depending on the type of biotic stress and the time at which the stress is imposed. The most common changes were seen in photosynthesis which is modified differently by rice plants to conserve energy, disrupt food supply for biotic stress agent, and initiate defense mechanisms or by biotic stressors to facilitate invasion and acquire nutrients, depending on their feeding style. Studies also provide evidence for the correlation between reactive oxygen species (ROS) and photorespiration and photosynthesis which can broaden our understanding on the balance of ROS production and scavenging in rice-pathogen interaction. Variation in the generation of phytohormones is also a key response exploited by rice and pathogens for their own benefit. Proteomics and metabolomics studies in resistant and susceptible rice cultivars upon pathogen attack have helped to identify the proteins and metabolites related to specific defense mechanisms, where choosing of an appropriate method to identify characterized or novel proteins and metabolites is essential, considering the outcomes of host-pathogen interactions. Despites the limitation in identifying the whole repertoire of responsive metabolites, some studies have shed light on functions of resistant-specific SMs. Lastly, we illustrate the potent metabolites responsible for resistance to different biotic stressors to provide valuable targets for further investigation and application.

## Background

Due to their sessile nature, plants are exposed to various biotic and abiotic stresses. Biotic stress is induced by pathogenic bacteria, fungi, viruses, and nematodes; pest attacks; and invasion of parasitic plants. Abiotic stress is caused by adverse environmental conditions such as drought, excess salt, flood, extreme heat and cold, heavy metals, and radiation (McDowell and Dangl 2000; Sarwat et al. 2013). Pathogens and pests, which induce biotic stress, are responsible for significant yield losses in rice, of around 30.0% globally in 2019 (Savary et al. 2019); therefore, these stresses present a threat to food supply. The main goal of pathogens and pests is to obtain nutrients from plants; however, to achieve this, they cause disease and weaken the plant to enable easy access to obtain nutrients. Pathogens can be biotrophs, necrotrophs, or hemibiotrophs, based on the method of nutrient acquisition (Freeman and Beattie 2008). Plant parasitic nematodes are migratory or sedentary biotrophic obligate parasites, which feed on plant tissues by initiating special feeding structures or incorporating cell wall-degrading enzymes, virulence proteins (Ali et al. 2017; Sato et al. 2019). Insects, particularly herbivorous species, can be divided into chewing and piercing-sucking insects. Chewing insects break and chew plant tissues, resulting in mechanical damage to the plants. Conversely, piercing-sucking insects penetrate plant cells and obtain nutrients from vascular tissues (Fujita et al. 2013). Furthermore, invading nematodes, insects, and tools used for agricultural practices act as vectors and transmit viruses into host plants (Alexander and Cilia 2016).

In response to biotic stressors, plants have developed an array of dynamic constitutive and inducible defense mechanisms to protect themselves against the damage caused by invading pathogens. Cell walls, waxy epidermal cuticles, and barks serve as constitutive defense mechanisms, which, along with structural firmness, act as a first line of defense. Pathogen-associated molecular pattern (PAMP)-triggered immunity (PTI) and effector-triggered immunity (ETI) are inducible defense mechanisms (Bigeard et al. 2015), which have been explained using a “zigzag” model (Jones and Dangl 2006). Importantly, host plants activate intricate networks of signaling cascades associated with the generation of reactive oxygen species (ROS) and the activation of hormones. Additionally, these cascades regulate kinase signaling to induce defense-related genes via the activation of transcription factors (TFs). Consequently, various secondary metabolites (SMs) and antimicrobial compounds such as phytoalexins and phenolics, are synthesized (Jain et al. 2019).

Proteins and metabolites, the final genome products, are involved in fundamental life processes. To overcome biotic stress, plants utilize multiple classes of proteins, including: (1) catalytic enzymes involved in cell wall modifications, phytohormones, ROS, and pathogenesis-related (PR) proteins; (2) TFs and post-translational factors; and (3) receptors and receptor-like kinases (Wu et al. 2016; Wu et al. 2017; Meng et al. 2019). Meanwhile, plant metabolites have distinct functions. More than 200,000 plant metabolites (Kang et al. 2019) have been classified into three dominant groups: primary metabolites, secondary (or specialized) metabolites, and hormones, which have overlapping functions (Erb and Kliebenstein 2020). Furthermore the contribution of primary metabolites to cellular energy supply and structure, phytohormones, and SMs is also important (Jwa et al. 2006). Four well-characterized hormones, abscisic acid (ABA), salicylic acid (SA), jasmonates (JA), and ethylene, play a critical role in modulating cellular mechanisms and activating plant immunity (Verma et al. 2016). SMs include phenolics produced via the shikimic and malonic acid pathways, terpenes via the mevalonic acid (MVA) and 2-C-methylerythritol 4-phosphate (MEP) pathways, and nitrogen-containing compounds from nitrogen-containing amino acids (Cheah et al. 2020; Khare et al. 2020). SMs function as antimicrobial compounds, damage–associated molecular patterns (DAMPs), and virulence factors for pathogens and are involved in callose deposition and the regulation of programmed cell death (Piasecka et al. 2015; Zaynab et al. 2018). In rice, metabolites involved in defense against biotic stress include volatile indole, glucosinolates, benzoxazinoids, phenylpropanoid phytoalexins, diterpenoid phytoalexins, and phenylamides (Erb and Kliebenstein 2020). Accordingly, studying proteins and metabolites is critical to understand the sophisticated plants’ responses to different biotic stressors under the view of proteome and metabolome. Proteomics approach is used to detect and analyze proteins. This tool can identify wide array of proteins including observation of any change in protein level during specific developmental stage of plants or plants under stresses (Tan et al. 2017; Liu et al. 2019). Moreover, proteomics can reflect the metabolic processes and their possibilities to interact with important regulatory pathways. Metabolomics approach is used to detect metabolites which are the end products of different regulatory processes and elucidate the molecular mechanisms behind any kind of variation in plants more efficiently compared to the levels of transcripts and proteins (Arbona et al. 2013). This approach differs from other omics approaches as metabolites are highly complex and requires more than one analytical platform to analyze them efficiently (Salem et al. 2020).

Since 2010, studies have investigated global changes in the composition of proteins and metabolites in plants exposed to biotic stress (for review, see Draper et al. 2011; Kushalappa and Gunnaiah 2013; Sarwat et al. 2013; Feussner and Polle 2015; Alexander and Cilia 2016; Hong et al. 2016; Meena et al. 2017; Peyraud et al. 2017; Chen et al. 2019). These studies have expanded our understanding of the regulatory mechanisms underlying plant responses and the invasive success of pests (Parker et al. 2009; Okazaki and Saito 2016). More recently, studies conducted in rice, one of the most important cereal crops, have revealed responses common to other plants as well as those specific to rice. In this review, we summarize recent findings from the proteomics and metabolomics studies in rice upon attack of various biotic stress agents, including rice mutants with altered disease resistance (Table 1 and Table 2). Finally, we suggest a framework for the improvement of rice performance.

**Table 1:** Global proteomics studies investigating biotic stress responses in rice

**Method**	**Bacteria**	**Cultivars**	**Key finding**	**Reference**
2-DE, MALDI-TOF MS	*Xoo* races T7174 (IC) and Xo7435 (C)	Java 14	Thaumatin-like protein (PR5) and probenazole (PBZ) were triggered by JA	Mahmood et al. 2006
2-DE, MALDI-TOF/TOF MS	*Xoo* race PXO99A (IC) and DY89031 (C)	*Xa21*-transgenic suspension cells	Nine putative PM-associated proteins with potential functions in rice defense were identified	Chen et al. 2007
2-DE, MALDI-TOF-TOF MS	*Xoo* strain Zhe173 (IC)	somatic hybrid line SH76 (R)	Majority of DEPs were involved in photosynthesis	Yu et al. 2008
2-DE, NanoLC MS/MS	*P. fluorescens strain* KH-1	Co43	*P. fluorescens* modulated rice metabolic pathways including energy metabolism and defense	Kandasamy et al. 2009
2-DE, MALDI-TOF MS	*Xoo* race Xo7435	Thaumatin-like protein gene transgenic-OX line	Variation in oxidative stress and energy metabolism associated proteins was observed in disease resistance	Mahmood et al. 2009a
2-DE, MALDI-TOF MS	*Xoo* races T7174 (IC) and Xo7435 (C)	Java 14 treated with probenazole	PR5 was highly induced in PBZ pretreated plants during their interaction with *Xoo*	Mahmood et al. 2009b
2-DE, MALDI-TOF MS	*Sinorhizobium meliloti* 1021	NPB	Defense related proteins were highly induced in root, whereas photosynthesis related proteins induced in leaf	Chi et al. 2010
LC-MALDI-MS/MS	*Xoo* XKK.12	Baldo	Virulence- associated factors were identified	González et al. 2012
2-DE, MALDI-TOF-MS	*Xoo* strain 89,773–1-1	9311	Disease resistance signal transduction, pathogenesis, and regulation of cell metabolism were activated	Li et al. 2012a
2-DE, MALDI-TOF MS and nESI-LC-MS/MS	*Xoo* strain K3	Dongjin	DUF26, β-1,3-glucanase, and basic secretory protein family proteins as the main host defense related proteins	Wang et al. 2013
LC-MS/MS	*Xoo* strain Zhe173	IRBB5 (R)	Several epigenetic factors regulated plant disease resistance pathway by alternating phosphorylation and dephosphorylation	Hou et al. 2015
2-DE and MALDI-TOF MS	*Xoo* isolate DX133	PB1 (S) and *O. longistaminata* (R)	Proteins related to defense response were mainly found expressed in the resistant plants	Kumar et al. 2015
2D-DIGE, MALDI-TOF-MS	*Xoo* strain PXO124	*O. meyriana*	Peroxidase was critical in the early response of *O. meyriana*	Chen et al. 2016
**Method**	**Fungi**	**Cultivars**	**Key finding**	**Reference**
2-DE, MALDI-TOF-MS	*M. oryzae* race KJ401 (IC) and KJ101 (C)	Jinheung	Receptor-like protein kinases, pathogenesis-related proteins, and JA were induced in incompatible interaction	Sun et al. 2004
2-DE, ESI Q-TOF MS	*Rhizoctonia solani* strain LR 172	Labelle (S) and LSBR-5 (R)	3- β-hydroxysteroid dehydrogenase/isomerase was first identified in resistant rice	Lee et al. 2006
2DE, MALDI-TOF MS	*M. oryzae* isolate Hoku1	ZTS (S) and ZTR (R, carryig *Pi-zt*)	Whole plant-specific resistance was associated with thaumatin-like protein	Koga et al. 2012
2-DE, MALDI-TOF/TOF MS	*M. oryzae* race ZC_13_ isolate 97-151a	CO39 (S) and C101LAC (R)	Resistant cultivar was more sensitive to SA signaling system	Li et al. 2012b
2-DE, MALDI-TOF-MS or nESI-LC-MS/MS	*M. oryzae* race KJ401 and KJ301	Jinheung	Different defense responses of rice and pathogenicity of *M. oryzae*	Kim et al. 2013
2-DE, MALDI-TOF/TOF-MS and nESI-LC-MS/MS	*Cochliobolus miyabeanus* strain SHS-2	Dongjin	Enzymes involved in the Calvin cycle and glycolysis were decreased; but the TCA cycle, amino acids, and ethylene biosynthesis were increased	Kim et al. 2014a
2-DE, MALDI-TOF/TOF and nanoLC-MS/MS	*M. oryzae* race ZC_13_	CO39 (S) and C101LAC (R)	Resistant rice had more and rapid signal transduction cascades	Li et al. 2015
iTRAQ, LC-MS/MS	*M. oryzae* ZHONG-10-8-14	NPB	Activation of ABA signaling in the early stage of infection, but CK signaling in later stages of infection	Cao et al. 2016
2-DE, MALDI-ToF	*R. solani* (WGL-12-1) of AG-1 IA	Four susceptible and two tolerant cultivars	Novel factors associated with susceptibility and resistance	Prathi et al. 2018
iTRAQ, HPLC-MS/MS	*M. oryzae* isolates KJ201 and RB22	NPB (WT) and NPB-Piz-t	Seven common proteins induced by *Piz-t* compatible and incompatible interactions	Tian et al. 2018
2-DE, MS/MS	*M. oryzae*	14 rice varieties	Functional correlation between nuclear reprogramming and immune response during blast disease	Narula et al. 2019
iTRAQ, LC-MS/MS	*R. solani* isolate AG1 IA	Lemont (S) and Teqing (R)	A network of SA, JA, ROS, and the TCA cycle related proteins conferred resistance against *R. solani*	Ma et al. 2020a
iTRAQ, HPLC-MS/MS	*M. oryzae* isolates Guy-11 and YN716	VP-1636 (WT) and GN-5 (*pi21-*mutant)	JA, SA, and ethylene metabolisms were upregulated in mutant line	Nawaz et al. 2020
2-DE, LC-MS/MS	*M. oryzae* (race 007.0), and its elicitor chitin	NPB and suspension cell derived from NPB calli	Rapid alkalinization factors, phytosulfokines, and novel immune response peptide were identified	Wang et al. 2020
**Method**	**Virus**	**Cultivars**	**Key finding**	**Reference**
2-DE, MALDI-TOF/TOF-MS	Rice black-streaked dwarf virus	Huai 5 (S)	Overaccumulation of H_2_O_2_ disrupted photosynthesis and metabolism and caused oxidative stress and abnormal plant growth	Xu et al. 2013
2-DE, MALDI-TOF MS	Rice stripe virus (RSV)	Wuyujing 3 (S) and Xudao 3 (R)	Downregulation of heat shock protein, protein disulfide isomerase, glyoxalase in Wuyuing 3	Yang et al. 2013
iTRAQ and RP-HPLC, LC-MS/MS	RSV	Wuyujing 3	Changes of chlorosis, cell death and plant defense by RSV	Wang et al. 2015
1-DE, LC-MS/MS	Southern rice black-streaked dwarf virus	NPB treated with cytosinpeptidemycin	PR and HSP were triggered by cytosinpeptidemycin	Yu et al. 2018
**Method**	**Insect**	**Cultivars**	**Key finding**	**Reference**
iTRAQ, nano-LC ESI QqTOF MS	BPH, *Nilaparvata lugens* Stål)	TN1 (S) and TN1 carrying *Bph15* (R)	Glycine cleavage system protein was upregulated in the resistant lines.	Wei et al. 2009
2D-DIGE; and 2-DE, MALDI TOF/TOF-MS	SBPH, *Laodelphax striatellus* Fallén)	Rice lines 02428 (S) and Pf9279–4 (R)	ROS scavenging and SA-mediated SAR were more active in resistant rice	Dong et al. 2017
iTRAQ and LC-MS/MS	BPH	PS (*Indica*, S), PR (*O. officinalis*, R), and their hybrid line HR	SMs, carbon metabolism, and glyoxylate and dicarboxylate metabolism were key markers of resistance	Zhang et al. 2019
nano-LC-MS/MS	*Cnaphalocrocis medinalis*	TN1 (S) and Qingliu (R)	Phenylalanine ammonia lyase and chalcone synthase were higher in resistant rice	Cheah et al. 2020
iTRAQ and nano-LC-MS/MS	BPH Biotype I and Biotype Y	TN1 (S) and YHY15 (moderately-R)	Post-translational modifications, protein turnover, and chaperones presented a significant difference between two BPH biotypes	Zha and You 2020
**Method**	**Nematode**	**Cultivars**	**Key finding**	**Reference**
HPLC-MS/MS	*Meloidogyne graminicola*	NPB and Khao Pahk Maw	α-linolenic acid, glutathione, and phenylpropanoid biosynthesis were involved in resistance	Xiang et al. 2020
**Method**	**Others**	**Cultivars**	**Key finding**	**Reference**
2-DE, MS/MS	NA	Kinmaze (WT) and LMM *cdr2*	Active metabolic changes were associated with programmed cell death	Tsunezuka et al. 2005
2-DE, MALDI-TOF-MS	NA	LMMs *spl1*	PBZ1 served as a cell death marker and defense protein	Kim et al. 2008
2-DE, MALDI-TOF/TOF	NA	Zhefu802 (WT) and LMM *spl5*	Defense response was induced, and amino acid metabolism and photosynthesis were reduced in *spl5*	Chen et al. 2013
2-DE, MALDI-TOF/TOF MS	NA	CO39 (S) and C101LAC (R) treated with Me-JA	MeJA induced higher ROS in resistant rice	Li et al. 2014
LC-MS/MS	NA	ZH11 (WT) and LMM *oscul3a*	Differentially expressed proteins were chloroplast and cytoplasm proteins	Gao et al. 2019
2-DE, MALDI-TOF/TOF MS and NanoLC-MS/MS	NA	CO39 (S) and C101LAC (R, carrying *Pi-1* gene) treated with SA	Phosphorylation regulation by SA contributed differently in resistant and susceptible rice	Sun et al. 2019

Abbreviations: *Xoo Xanthomonas oryzae* pv. *oryzae*, *IC* Incompatible, *C* Compatible, *PM* Plasma membrane, *S* Susceptible, *R* Resistant, *DEP* Differentially expressed proteins, *P. fluorescens Pseudomonas fluorescens*, *OX* Over-expression, *NPB* Nipponbare, *WT* Wild type, *PB1* Pusa Basmati1, *M. oryzae Magnaporthe oryzae*, *ABA* Abscisic acid, *CK* Cytokinin, *SA* Salicylic acid, *JA* Jasmonic acid, *ROS* Reactive oxygen species, *HSP* Heat shock protein, *TCA* Tricarboxylic acid, *BPH* Brown plant hopper, *TN1* Taichung Native-1, *SM* Secondary metabolite, *SBPH* Small brown planthopper, *SAR* Systemic acquired resistance, *LMM* Lesion mimic mutant, *1-DE* One-dimensional gel electrophoresis, *2-DE* Two-dimensional gel electrophoresis, *2D-DIGE* Two-dimensional difference gel electrophoresis, *MS* Mass spectrometry, *MALDI-TOF* Matrix-assisted laser desorption ionization time of flight, *TOF* Time-of-Flight, *Q-TOF* Quadrupole time-of-flight, *QqTOF* Quadrupole-quadrupole-time-of-flight, *iTRAQ* Isobaric tags relative and absolute quantification, *LC-MS* Liquid chromatography mass spectrometry, *HPLC* High-performance liquid chromatography, *RP-HPLC* Reverse phase-high performance liquid chromatography, *UHPLC* Ultra high performance liquid chromatography, *GC-MS* Gas chromatography mass spectrometry, *CE-MS* Capillary electrophoresis mass spectrometry, *nESI* Nano-electrospray ionization source, *NA* Not applicable

**Table 2:** Global metabolomics studies investigating biotic stress responses in rice

**Method**	**Bacteria**	**Cultivars**	**Key finding**	**Reference**
LC-TOF-MS and GC-TOF-MS	*Xoo* PXO99 and ∆PXO994rax- ST	TP309 (S) and TP309_Xa21	Alkaloid biosynthesis was increased specifically in TP309_Xa21 to PX099 but not ∆PXO994rax- ST	Sana et al. 2010^a^
RP-HPLC-MS	*Azospirillum* strains 4B and B510	Cigalon and NPB	Phenolic compounds were mainly affected	Chamam et al. 2013
LC-MS	*Burkholderia glumae* AU6208 and *Escherichia coli* B6	Cigalon and NPB treated with *Azospirillum lipoferum* 4B	Flavonoid compounds and hydroxycinnamic acid (HCA) derivatives changed differently upon each bacterium	Chamam et al. 2015
LC-MS/MS	*Xoo*	Basmati 385 treated with *Pseudomonas aeruginosa* BRp3	Rice defense-related enzymes were activated by *P. aeruginosa*	Yasmin et al. 2017
HPLC	*Pseudomonas putida* RRF3	TKM 9	*P. putida* stimulated plant defense responses and altered rhizosphere chemical constituents	Kandaswamy et al. 2019
UHPLC-QE Orbitrap/MS	*Bacillus pumilus* LZP02	Longgeng 46	*Bacillus pumilus* enhanced carbohydrate metabolism and phenylpropanoid biosynthesis	Liu et al. 2020
UHPLC-DAD/ESI-QTOF	10 PGPR strains and *B. glumae* AU6208	NPB	Common metabolomics signature of nine compounds as rice response to different PGPR	Valette et al. 2020
**Method**	**Fungi**	**Cultivars**	**Key finding**	**Reference**
FIE-MS, GC-TOF-MS	*M. oryzae* strain Guy11	*B. distachyon* ABR1, *H. vulgare* Golden Promise and CO39	Common metabolic re-programming strategy was deployed by *M. oryzae* in different hosts	Parker et al. 2009
HPLC-MS/MS	*Fusarium fujikuroi* strain VE13	Dorella (S) and Selenio (R)	Sakuranetin accumulated in resistant cultivar	Siciliano et al. 2015^a^
GC-MS	*Harpophora oryzae* strain R5–6-1 and *M. oryzae* strain Guy11	CO39	Different induction patterns of metabolites of the shikimate and lignin against pathogenic and mutualistic fungi	Xu et al. 2015
GC-MS	*R. solani*	*Narayan* with and without *Bacillus amyloliquefaciens* (SN13) treatment	Identified novel aspect of rare sugar induced by *Bacillus amyloliquefaciens*	Srivastava et al. 2016
CE/TOF-MS	*R. solani* AG-1 isolate C-154	29S (S) and 32R (R)	Canavanine was significantly higher in resistant rice	Suharti et al. 2016a^a^
CE/TOF-MS	*R. solani* AG-1 isolate C-154	29S (S) and 32R (R)	Chlorogenic acid specifically induced in resistant rice	Suharti et al. 2016b^a^
CE/TOF-MS	*R. solani* AG-1 isolate C-154	29S (S) and 32R (R)	Distinct responses of susceptible and resistant rice	Suharti et al. 2016c^a^
GC-MS	*R. solani* AG1-IA isolate BRS1	PB1 (S)	Altered carbon metabolism and perturbed hormonal signaling	Ghosh et al. 2017
2-DE, MALDI-TOF MS/MS & GC-MS	*R. solani* isolate AGI-IA	IR-64 (WT) and *AtNPR1*-OX line	Novel immunity-related prognostic proteins induced by *AtNPR1*	Karmakar et al. 2019^a,b^
QTOF-UPHPLC MS	*M. oryzae* strain Guy11	CO39, NPB, and LTH (S); Pi-gm, Pi-4B, and Pi-B (R)	Bayogenin 3-O-Cellobioside, a saponin compound, was first identified in rice for the first time	Norvienyeku et al. 2020^a^
**Method**	**Insect**	**Cultivars**	**Key finding**	**Reference**
^1^H NMR	BPH	TN1 (S) and B5 (R)	Activation of GABA shunt and shikimate metabolisms was vital for BPH resistance	Liu et al. 2010^a^
GC-MS	Rice gall midge biotype 1 (GMB1)	TN1, Kavya, and RP2068	Potential biomarkers of rice-gall midge interaction were identified	Agarrwal et al. 2014^a^
GC-MS	GMB1	RP2068-18-3-5 (R)	During HR, upregulation of LPO and LPO marker metabolite azelaic acid; and higher accumulation of GABA at the feeding site	Agarrwal et al. 2016^a^
UHPLC-MS and GC-MS	Rice stem borer (*Chilo suppressali*)	Minghui 63	Activation of phytohormones and shikimate-mediated and terpenoid-related secondary metabolism	Liu et al. 2016
GC-MS	BPH	TN1 (S) and YHY15 (R)	Resistance to BPH was mediated by SM synthesis through the shikimate pathway	Peng et al. 2016^a^
^1^H NMR and GC-FID/MS	BPH	TN1 (S) and NIL-*Bph15* (R)	BPH adapts and recovers at different stage in susceptible and resistant plants	Liu et al. 2017
UPLC-Q-TOF MS	BPH	Dongjin treated with *B. velezensis* YC7010	*B. velezensis* induced SA, JA, and secondary metabolites to enhance resistance	Harun-Or-Rashid et al. 2018
GC-MS	BPH	NPB (S) and *Bph6*-transgenic line R6 (R)	*Bph6* resistance gene affected lipid levels in leaf sheath only	Zhang et al. 2018^a^
GC-MS & LC-MS	BPH	TN1 (S), IR36 and IR56 (R)	Defense-related metabolites, cyanoamino acids, and lipid metabolism were increased by BPH and were more stable in resistant cultivars	Kang et al. 2019^a^
UPLC-QToF-MS	BPH	KDML105 (S) and IL308 (R)	Susceptible and resistant rice induced common SMs at different levels	Uawisetwathana et al. 2019^a^
LC-ESI-MS/MS	*Cnaphalocrocis medinalis*	Minghui 63	JA-dependent signaling pathway was found vital in response to leaf folder	Wang et al. 2020
**Method**	**Nematode**	**Cultivars**	**Key finding**	**Reference**
HPLC	*Ditylenchus angustus*	Two susceptible and five resistant cultivars	Induction and accumulation of phenolic compounds in the resistant varieties	Gill et al. 1996^a^
**Method**	**Others**	**Cultivars**	**Key finding**	**Reference**
LC-MS and Q-TOF MS/MS	NA	ZH17 (WT), *wrky62, wrky76* and dsOW62/76	SA, JA, and phenolamides were increased and free pools of flavonoids were decreased in the double mutant	Liang et al. 2017

Abbreviations: single asterisk “^a^” indicates comparative metabolomics studies of resistant and susceptible plants; “^b^” indicates the study applied both proteomics and metabolomics approaches; *Xoo Xanthomonas oryzae* pv. *oryzae*, *S* Susceptible, *R* Resistant, *WT* Wild type, *OX* Over-expression, *NPB* Nipponbare, *PB1* Pusa Basmati1, *PGPR* Plant growth promoting rhizobacteria, *B. glumae Burkholderia glumae*, *M. oryzae Magnaporthe oryzae*, *B. distachyon Brachypodium distachyon*, *H. vulgare Hordeum vulgare*, *SA* Salicylic acid, *JA* Jasmonic acid, *BPH* Brown plant hopper, *TN1* Taichung Native-1, *HR* Hypersensitive response, *LPO* Lipid peroxidation, *SM* Secondary metabolite *NIL* Near-isogenic line, *B. velezensis Bacillus velezensis*, *LMM* Lesion mimic mutant, *dsOW62/76* Mutant containing RNA interfering constructs of *OsWRKY62* and *OsWRKY76*, *2-DE* Two-dimensional gel electrophoresis, *MS* Mass spectrometry, *MALDI-TOF* Matrix-assisted laser desorption Ionization time of flight, *TOF* Time-of-Flight, *Q-TOF* Quadrupole time-of-flight, *LC-MS* Liquid chromatography mass spectrometry, *HPLC* High-performance liquid chromatography, *RP-HPLC* Reverse phase-high performance liquid chromatography, *UHPLC* Ultra high performance liquid chromatography, *GC-MS* Gas chromatography mass spectrometry, *CE-MS* Capillary electrophoresis mass spectrometry, ^*1*^*H NMR* Proton nuclear magnetic resonance, *FID* Flame-ionization detection, *DAD* Diode array detector, *NA* Not applicable

## Methods Used to Study Plant Proteomics and Metabolomics

Proteomics and metabolomics analyses involve a variety of techniques. For example, proteomics studies can involve gel-based or gel-free techniques. Gel-based methods are the most commonly used for global protein analyses, and include two-dimensional gel electrophoresis (2-DE) and difference gel electrophoresis (DIGE). In combination with advanced mass spectrometry (MS) techniques, hundreds of proteins can be detected in a single polyacrylamide gel, allowing analyses of their mass-to-charge ratio and post-translational modifications (Vanderschuren et al. 2013; Tan et al. 2017). Gel-free techniques were developed to address the limitations associated with gel-based techniques, such as reproducibility, bias, the need for technical expertise, and difficulties in detecting proteins present at low abundance or those that are highly acidic or basic (Tan et al. 2017). This approach utilizes three labeling methods: tag-based labeling such as isotope-coded affinity tag (ICAT), isobaric tags relative and absolute quantification (iTRAQ), tandem mass tag (TMT), and dimethyl and ^18^O labeling; metabolic labeling such as stable isotope labeling by amino acids in cell culture (SILAC) and ^15^N labeling; and label-free techniques, which use multi-dimensional capillary liquid chromatography (LC) coupled to nano-electrospray ionization-tandem mass spectrometry (NS-ESI-MS/MS) methods, such as sequential window acquisition of all theoretical mass spectra (SWATH-MS) (Tan et al. 2017; Ludwig et al. 2018).

Progression in analytical chemistry has led to the development of a range of metabolomics techniques, including gas chromatography (GC), liquid chromatography (LC), and capillary electrophoresis (CE) in combination with mass spectrometry (MS) and nuclear magnetic resonance (NMR) spectroscopy (Fukusaki and Kobayashi 2005; Piasecka et al. 2019). GC-MS is a popular method capable of quantifying the levels of volatile and semi-volatile organic compounds from diverse samples. Conversely, LC-MS is a more comprehensive method, and crude extracts can be used to quantify a wide variety of metabolites. Over time, LC-MS has been optimized, allowing the collection of more effective metabolomics data by introducing ultra-performance liquid chromatography coupled with high-resolution mass analysis methods, such as time-of-flight, Fourier transform, and Orbitrap-based MS (Salem et al. 2020). Another powerful technique is CE-MS; however, this is rarely used to analyze plant metabolites because of the time-consuming and diverse extraction requirements. However, this highly sensitive technique can classify metabolites into classes that other techniques cannot, particularly highly charged metabolites (Fukusaki and Kobayashi 2005; Salem et al. 2020). NMR spectroscopy is considered to be less biased due to its independence of ionization. In addition, this method is highly reproducible, requires minimum sample preparation, and can identify novel compounds (Valentino et al. 2020). NMR is rarely used to study rice metabolomics in response to biotic stress (Table 2). Proteomics and metabolomics require the use of multiple analysis tools and databases to efficiently detect and classify proteins and metabolites based on their specific functions and related pathways. Different analysis tools, as well as their specificities, have been well documented (Piasecka et al. 2019; Sarim et al. 2020). Commonly used databases include Gene Ontology knowledgebase (http://geneontology.org/), protein database of the National Center for Biotechnology Information, RiceCyc (http://pathway.gramene.org/gramene/ricecyc.shtml), OryzaCyc in the Plant Metabolic Network database (www.plantcyc.org), and Kyoto Encyclopedia of Genes and Genomes (http://www.genome.jp/kegg/).

## Response of Rice to Various Biotic Stresses Considering Proteomics and Metabolomics

Transcriptomics studies of rice have provided a wealth of information and a global view of mixed gene regulation in response to various biotic stressors (Anderson and Mitchel-Olds 2011). With the emergence of advanced methods for the validation of proteins and metabolites, progress has been made in elucidating the subsequent systematic changes that follow transcription in rice (Table 1 and Table 2).

### Response of Rice to Bacteria

*Xanthomonas oryzae* pv. *oryzae* (*Xoo*) causes bacterial blight, which is the most important disease of rice caused by bacterial pathogens. Studies investigating global changes in rice proteins in response to *Xoo* infection have been performed. Central carbon catabolism is reduced, whereas signal transduction associated with disease resistance, pathogenesis, and the regulation of cell metabolism are upregulated, including several putative resistance (R) genes, putative receptor-like kinases, and PR (Mahmood et al. 2006; Yu et al. 2008; Mahmood et al. 2009b; Sana et al. 2010; Li et al. 2012a). Particularly, thaumatin-like protein (PR5), probenazole (PBZ), Domain of Unknown Function 26 (DUF26), and β-1,3-glucanase were reported as the key findings in the early studies (Mahmood et al. 2006; Wang et al. 2013). A secretome analysis against *Xoo* identified virulence-associated factors and plant-specific proteins such as proteases or peptidases and proteins involved in host defense, the transport system, and maintaining redox balance (González et al. 2012; Wang et al. 2013; Kim et al. 2014b). A similar analysis was performed using a suspension of *Oryza meyeriana,* a wild species that is strongly resistant to *Xoo.* Upregulation of the signal transduction protein, LysM receptor-like kinase and defense protein, and downregulation of a ROS enzyme (peroxidase) and cell wall modifications (via expansins and pectin acetylesterase) were reported (Chen et al. 2016). Besides, phosphosites identification suggested that phosphorylation of TFs, kinases, epigenetic controlling factors, and disease resistant proteins may be functionally relevant to *Xoo* resistance in IRBB5 (Hou et al. 2015). Furthermore, a metabolomics study revealed differences between resistant and susceptible phenotypes against *Xoo* in the accumulation of metabolites before and after infection (Sana et al. 2010). Particularly, XA21-expressing plants differed from the wild-type (WT) plants, with higher levels of sugar alcohols, tricarboxylic acid cycle (TCA) intermediates, and miscellaneous compounds in the absence of treatment. After treatment, XA21 plants contained more responsive metabolites, including rutin, pigments, fatty acids and lipids, and arginine, which are likely required for polyamine biosynthesis and alkaloid metabolism. Notably, the virulence signal acetophenone was depressed in XA21.

Plant growth-promoting rhizobacteria (PGPR), whose growth is stimulated by root exudates, assist plants via nutrient uptake and phytohormone production. In proteomics analysis, photosynthesis and defense related proteins were found to accumulate by *Pseudomonas fluorescens* and *Sinorhizobium meliloti* (Kandasamy et al. 2009; Chi et al. 2010). Metabolite profiling was first performed in 2013, when two rice cultivars were infected with rice-associated *Azospirillum* species (*Azospirillum lipoferum* 4B and *Azospirillum* sp. B510). In that study, changes in phenolic compounds, such as flavonoids and hydroxycinnamic derivatives, were found to differ depending on the cultivar-PGPR strain interaction (Chamam et al. 2013). Additionally, Nipponbare inoculated with 10 PGPR strains presented common metabolomics signatures, including reduced alkylresorcinol [5-tridecyl resorcinol, 5-pentadecyl resorcinol, 5 (12-heptadecyl) resorcinol] levels and the differential induction of two antimicrobial compounds, N-p-coumaroylputrescine and N-feruloylputrescine, but in different manners (Valette et al. 2020). *Pseudomonas* is a PGPR used as a biocontrol agent against rice disease, due to its antagonism towards other bacteria and fungi. Analysis of roots and root exudates of rice infected with *Pseudomonas putida* by High-performance liquid chromatography (HPLC) revealed the induction of SA (Kandaswamy et al. 2019). In another study, *Pseudomonas aeruginosa* was found to produce compounds associated with systemic acquired resistance (SAR), including siderophores (1-hydroxy-phenazine, pyocyanin, and pyochellin), and antibacterial compounds, including 4-hydroxy-2-alkylquinolines and rhamnolipids (Yasmin et al. 2017). This newly isolated strain can also induce defense-related enzymes in rice plants, suggesting that it may have potential for improving rice plant performance against pathogens.

### Response of Rice to Fungi

*Magnaporthe oryzae*, the causal agent of rice blast disease, has caused huge losses in rice (Dean et al. 2012). Recently, proteomics and metabolomics studies investigating rice response to *M. oryzae* infection were reviewed (Meng et al. 2019; Azizi et al. 2019). In addition to the influence of *M. oryzae* on the basic biological processes of the host, such as photosynthesis and primary metabolism, global studies over the past decade have elucidated the detailed interaction between rice and *M. oryzae* considering whole proteins and metabolites (for review, see Azizi et al. 2019; Meng et al. 2019). Accordingly, metabolomics studies emphasize the difference between biotrophic and necrotrophic stages, such as the accumulation of metabolic photosynthetic sinks at biotrophic stage or phenolic compounds at necrotrophic stage (for review, see Azizi et al. 2019). In addition, a class of SMs also help to prevent fungal invasion via the antimicrobial activity of phytoalexins, including N-benzoyl tryptamine, N-cinnamoyl tryptamine, sakuranetin, and phenylamides, or the ROS-scavenging activity of serotonin. The induction or suppression of these compounds is tightly related to phytohormones such as ABA, JA, SA, and auxin. Furthermore, proteomics studies have revealed the roles of DUF26, nucleotide binding-leucine rich repeat, PRs, ROS production-scavenging enzymes, heat shock proteins (HSPs), nuclear reorganization-related proteins, TFs, and phytohormone signaling in rice resistance, whereby proteins related to pathogen perception and signal transduction are important during the early stage of infection (for review, see Meng et al. 2019). A recent study using iTRAQ found that probenazole-inducible protein 1 (PBZ1) and phenylpropanoid accumulated in both resistant and susceptible cultivars, which was in contrast to reports from previous studies utilizing the 2DE approach (Ma et al. 2020b). Interestingly, a metabolomic assay using HPLC identified a rice saponin, Bayogenin 3-O-cellobioside, which is the first saponin found in rice (Norvienyeku et al. 2020). Accordingly, the accumulation of Bayogenin 3-O-cellobioside is well corelated with blast resistance. Thus, improvements in these methods provide a more precise view of the interaction between rice and rice blast fungus on a case-by-case basis.

*Rhizoctonia solani* is a necrotrophic fungus that causes sheath blight in rice. In contrast to *M. oryzae*, this fungus causes cell death from the early stage of infection; thus, studies of *R. solani* infection have identified many distinct changes. The most notable changes have been observed in photosynthesis and sugar metabolism. The reduction of Ribulose-1,5-bisphosphate carboxylase/oxygenase (RuBisCo) large subunits was observed in an early study (Lee et al. 2006). Two metabolomics studies reported the induction of glycolysis and TCA cycle intermediates (succinate, pyruvate, and aconitate) and decreased levels of sugar metabolites (sucrose, glucose, fructose, glucosone, galactose, hexopyranose, turanose, maltose, and glucopyranose), suggesting that respiration in the infected tissue was enhanced for energy production. ROS, SA, JA, aromatic aliphatic amino acids, and phenylpropanoid intermediates also accumulated, accompanied by the suppression of myo-inositol, indicating the loss of antioxidant activity, which is consistent with the formation of lesions by necrotrophic pathogens (Suharti et al. 2016b; Ghosh et al. 2017). The increase of SA, known as an inducer of defense responses against biotrophic pathogens, is consistent with the following research which found the hemobiotrophic nature of *R. solani* (Kouzai et al. 2018). Global proteome and metabolome studies also suggested the distinct responses of resistant and susceptible phenotypes. Resistant cultivars were found to produce higher levels of antifungal proteins (β-1–3 glucanase and chitinase), ROS scavenging machinery, and 3β-hydroxysteroid dehydrogenase (3β-HSD) for the synthesis or regulation of plant steroids. Additionally, 14–3-3 protein, which is involved in protein interactions, and the chaperonin 60 β precursor were reduced (Lee et al. 2006). Glycolysis or gluconeogenesis and fatty acid β-oxidation for adenosine triphosphate (ATP) production were upregulated, whereas energy consumption was reduced in resistant plants via the reassimilation of photorespiratory ammonia or the regulation of energy metabolism. A high abundance of proteins related to glycolysis, α-amino acid biosynthesis, and stress response have also been observed in *R. solani* resistance through the analysis of transgenic rice expressing *AtNPR1*, a key regulator of SAR (Karmakar et al. 2019). Consistent with JA, lignification and signaling were found to be stable in resistant plants (Suharti et al. 2016c). Additionally, differences in ROS regulation between resistant and susceptible plants were noted (Ma et al. 2020a). Consistent with this, pipecolic acid, which regulates SAR and induces necrotic symptoms, was upregulated in susceptible plants (Suharti et al. 2016b). However, some studies reported unexpected findings, including the downregulation of Casparian strip membrane domain-like protein 2B1, which is passively required for lignin deposition in resistant cultivars, and the upregulation of spermidine hydroxycinnamoyl transferase 1, which is involved in the biosynthesis or modification of alkaloids, terpenoids, and phenolics in susceptible plants (Prathi et al. 2018). A study investigating protein changes after infection with another necrotrophic fungus, *Cochliobolus miyabeanus,* which causes brown spot disease in rice, suggested a pattern similar to that seen in response to *R. solani* infection (Kim et al. 2014a). Proteins involved in the Calvin cycle (fructose bisphosphate aldolase, sedoheptulose-1, 7-bisphosphatase, and RuBisCO) were reduced; however, oxaloacetate aspartate and aminotransferase, which are required for amino acid biosynthesis, and enzymes involved in redox homeostasis (peroxiredoxins, glutathione reductase, and NADP-dependent isocitrate dehydrogenase) were found to be accumulated.

Those studies in necrotrophic pathogens suggested a distinct response compared with hemibiotrophic pathogens at an early stage, which was exemplified by the reduction in photosynthesis, increase in energy production, and accumulation of ROS. However, current data from a limited number of proteomics and metabolomics studies indicate that different cultivars may use different mechanisms to respond to fungal pathogens.

### Response of Rice to Virus

Viruses transmitted to rice by either plants or insects induce physiological changes such as suppressed photosynthesis and chlorosis (for review, see Alexander and Cilia 2016). The key changes introduced by viral infection through insects as the vector are those involving carbon metabolism. Glyceraldehyde-3-phosphate dehydrogenase (GAPDH), a glycolytic enzyme, has emerged as a multifunctional protein in several non-metabolic processes and is increased in several plant species following viral infection (for review, see Alexander and Cilia 2016; Chen et al. 2013). Additionally, fluctuations in amino acids, which provide materials for viral replication, plant defense, ROS accumulation for chlorotic damage, and respiration in terms of energy supply, are also important responses to viral infection. These findings have been confirmed and clarified by proteome and metabolome studies. For example, chloroplast proteins are degraded, chlorophyll *a* and chlorophyll *b* synthesis is inhibited, and 26S proteasome is enhanced during rice stripe virus (RSV, *Tenuivirus* genus) infection (Wang et al. 2015). Furthermore, enhanced H_2_O_2_ production by rice black-streaked dwarf virus (RBSDV, *Reoviridae* family, *Fijivirus* genus) may diminish light absorption, resulting in reduced photosynthesis (Xu et al. 2013). Studies on uninfected and virus-infected rice, or on resistant and susceptible cultivars, have also identified a class of proteins and metabolites that underly viral resistance. Accordingly, RBSDV induces the production of ABA and cytokinins and reduces the production of indole-3-acetic acid, gibberellins, JA, and SA by suppressing expression of the related genes (Huang et al. 2018). In that study, gibberellic acid (GA) was able to rescue the typical dwarfing symptom, and pre-application of SA was able to reduce the severity of the disease. ROS, which has a dual function in chlorosis and in viral defense mechanisms, was found to accumulate at different levels in susceptible and resistant cultivars (Xu et al. 2013). In line with this, treatment with the antiviral bioactive SM, cytosinpeptidemycin, was shown to upregulate peroxidase, superoxide dismutase, and catalase in response to southern black-streaked dwarf virus infection (Yu et al. 2018). Additionally, the increased production of PR proteins, including PR5, PR10, and Bet v1 allergen or HSPs, has also been associated with resistance to virus in rice (Yang et al. 2013; Wang et al. 2015; Yu et al. 2018).

### Response of Rice to Insects

Interactions between plants and insects include dynamic defense mechanisms in plants and weapons in insects to enable successful invasion. Plants avoid being eaten in two ways, both involving a dominant role of SMs: repelling ovipositing herbivores along with attracting enemies and causing herbivore mortality. Insects may overcome this by secreting effectors in salivary proteins or capitalizing SMs (Lu et al. 2018). Although the strategies used by plants to defend against each kind of insect may vary (Harun-Or-Rashid et al. 2018), common mechanisms involve JA signaling, detoxification, cell wall modifications, photosynthesis, phytohormones, and defensive SMs (for review, see Ling et al. 2019; Zogli et al. 2020).

The brown planthopper (BPH, *Nilaparvata lugens* Stål*,* Hemiptera: Delphacidae) is a typical monophagous vascular feeder. Proteomics and metabolomics studies on rice response to BPH infection have revealed the occurrence of dynamic changes. Lipid transport and metabolism, SM biosynthesis, amino acid transport and metabolism, and phytohormone signaling are commonly induced by BPH in both susceptible and resistant cultivars (Wei et al. 2009; Dong et al. 2017; Zhang et al. 2018; Zhang et al. 2019; Zha and You 2020). Notably, studies that have utilized resistant and susceptible cultivars to observe changes on protein and metabolite levels have identified markers and features associated with resistance to BPH infection. This has also been demonstrated in time-course studies during each stage of BPH infection. A metabolomics study in leaf sheath and honeydew revealed enhanced fatty acid oxidation, glyoxylate cycle, gluconeogenesis, and γ-aminobutyric acid (GABA) shunt in susceptible cultivars, whereas glycolysis was upregulated in resistant cultivars, resulting in the production of substrates for SM synthesis via the shikimate pathway (Liu et al. 2010; Peng et al. 2016). Lower levels of amino acids in nymphs at the early, but not the late, stage of infection were reported in resistant cultivars compared with susceptible cultivars, indicating the rapid adaptation of BPH (Liu et al. 2017). In another study, there were clear differences in the response of resistant and susceptible cultivars to small BPH (*Laodelphax striatellus* Fallén*,* Homoptera: Delphacidae) during the early stage, when the resistant cultivar displayed less tissue damage due to the upregulation of ROS removal machinery and SA (Dong et al. 2017). A lipidomics study reported that the resistance conveyed by *Bph6* gene involves wax biosynthesis (for example fatty acid methyl esters) (Zhang et al. 2018). Recently, Kang et al. (2019) reported that primary metabolism was inhibited in all cultivars at the early stage (24 h) but only recovered in the late stage (96 h) in resistant cultivars. In that study, amino acids, organic acids, and fatty acids were also found to be stable in resistant cultivars. Higher levels of flavonoid glycosides (schaftoside, iso-schaftoside, rhoifolin, and apigenin 6-C-α-l-arabinoside-8-C-β-l-arabinoside) were induced in resistant rice compared with susceptible rice (Uawisetwathana et al. 2019).

Phytohormones play an important role in the interaction between rice and hopper. The function of SA in this interaction appears ambiguous, as it has been reported to be upregulated in both susceptible (Peng et al. 2016) and resistant (Dong et al. 2017) cultivars. Enhanced JA metabolism is the main difference in the resistance of wild rice *Oryza officinalis* compared to BPH-susceptible *O. sativa* (Zhang et al. 2019). Interestingly, both SA and JA signaling was enhanced in the endophytic strain *Bacillus velezensis* YC7010, which induces resistance to BPH infection in rice (Harun-Or-Rashid et al. 2018).

Studies on the response of rice to other insects have also revealed specific responses. The response of a resistant rice line (cultivar Qingliu) to rice leafroller (*Cnaphalocrocis medinalis*) involved the activation of the Calvin cycle and the light reaction of photosynthesis, followed by the biosynthesis of amino acids and other metabolites (Cheah et al. 2020). Furthermore, resistance was determined by flavonoid biosynthesis at a specific rate and time. Rice also defends against the rice stem borer (*Chilo suppressalis*) using a similar mechanism. An integrated transcriptomics and metabolomics study suggested increased photosynthesis via the accumulation of monosaccharides and not of oligosaccharides, galactinol, and various amino acids (Liu et al. 2016). Conversely, the resistance of rice to rice gall midge was found to involve differences in fatty acids before and after infection, whereas glutamine and 23-oxotetracosanoic acids were associated with susceptibility (Agarrwal et al. 2014).

### Response of Rice to Nematodes

Nematodes are universally present in nature and include species that are parasitic to plants, including rice (Sato et al. 2019). Studies investigating rice-parasitic nematode interactions have generally involved mutants and transcriptome analyses, with a notable lack of proteomics and metabolomics studies. In 1996, a group of researchers used HPLC to evaluate differences in the phenolic profiles of five resistant and two susceptible deep-water rice upon *Ditylenchus angustus* infection. They reported changes in SMs, such as chlorogenic acids and phytoalexin sakuranetin, which were mainly identified in the resistant rice (Gill et al. 1996). A previous proteomics study on the rice and root-knot-nematode (*Meloidogyne graminicola*) interaction revealed new proteins as well as changes in existing proteins (Xiang et al. 2020). Importantly, proteins involved in stress, metabolic pathways, and SM biosynthesis were found to accumulate at the early stage of infection, and this continued to the later stage of infection. Additionally, an integration of transcript analyses revealed that four specific proteins related to α-linolenic acid metabolism, phenylpropanoid biosynthesis, glutathione metabolism, and plant–pathogen interaction pathways were downregulated in the susceptible cultivar but upregulated in the resistant cultivar (Xiang et al. 2020).

## Proteomics and Metabolomics Studies in Mutants with Altered Defense Response

Lesion mimic mutants have been used to study resistance for over two decades. Therefore, proteomics studies on lesion mimic mutants of rice have widened our understanding of the fundamentals of hypersensitive response (HR)-like symptom. A 2DE study on the lesion-mimic mutant Cell death and resistance 2 (*cdr2*) and Rice spotted leaf 5 (*spl5*) reported the accumulation of defense-related proteins, including probenazole-induced (PBZ1) protein (Tsunezuka et al. 2005; Chen et al. 2013). PBZ1 protein is also highly inducible in the Squamosa promoter-binding-like protein 1 (*spl1*) mutant (Kim et al. 2008). Regarding central metabolism, photosynthesis is inhibited whereas respiration is enhanced via the down and upregulation of the associated proteins, respectively. Interestingly, the overproduction of ROS in lesion mimic mutants induces ROS-scavenging enzymes, such as L-ascorbate peroxidase 7, but suppresses superoxide dismutase in *cdr2* mutant, confirming that the tight regulation of ROS is correlated with the formation time and density of lesions. In addition, pathogen-infected mimic responses, such as enhanced lipid metabolism, were found to suppress carbon and nitrogen metabolism and the accumulation of SA and SMs in *oscul3a* mutants (Gao et al. 2019).

Proteomics and metabolomics have been studied to understand PTI, also known as basal resistance. Loss-of-function of *Pi21*, a quantitative resistance gene encoding a proline-rich protein that includes a putative heavy metal-binding domain and putative protein-protein interaction motifs, results in non-race specific and durable blast resistance (Fukuoka et al. 2009). Protein profiling of a *Pi21*-knockout mutant in the absence of pathogen infection revealed the accumulation of photosynthates, carbohydrate metabolites, and small molecule metabolites, compared with the WT plants (Nawaz et al. 2020). Additionally, metabolomics studies have shown that the enhanced basal resistance associated with loss-of-function of WRKY62 and WRKY76 (dsOW62/76) is associated with the accumulation of amino acids, constituents of TCA, phenolic acids derived from the phenylpropanoid pathway, upregulated SA and JA, and antimicrobial phytoalexins, such as sakuranetin and phenolamides (Liang et al. 2017).

Proteomics has also been studied to understand ETI in *Pizt*-expressing plants in response to avirulent and virulent isolates, which suggested that various specific responses are induced by Pizt (Tian et al. 2018). Accordingly, fluctuations in 56 proteins were common between Pizt and WT plants after infection and included PR proteins, proteins related to hormonal regulation and defense and stress response, receptor-like kinases, and cytochrome P450. Interestingly, the incompatible interaction differed significantly from the compatible interaction in only a few proteins, including alcohol dehydrogenase I, receptor-like protein kinase, endochitinase, similar-to-rubisco large subunit, NADP-dependent malic enzyme, and two hypothetical proteins. This finding raises the question of whether variation in only those compounds could lead to different ETI outcomes.

## Conclusions and Prospects

### Common Metabolites and Specific Metabolites upon each Biotic Stress

Studies investigating the response to biotic stresses have commonly reported changes in photosynthesis, possibly due to the abundant related proteins and metabolites. Photosynthesis is upregulated or downregulated in susceptible or resistant phenotypes in response to different pathogens. In response to insects, two theories have been proposed to explain this phenomenon. The first notes that the intrinsic activation of photosynthesis provides organic compounds for the synthesis of defense-related metabolites as a result of pathogen manipulation for food resources (Cheah et al. 2020). An opposing theory states that plants suppress photosynthesis to conserve energy and reduce food supply to pathogens. Photosynthesis is enhanced by *Xoo* (Sana et al. 2010; Li et al. 2012a), insect (Liu et al. 2016; Cheah et al. 2020), and *M. oryzae* infection in the biotrophic stage (Azizi et al. 2019) but suppressed by the necrotrophic fungus *R. solani* (Lee et al. 2006; Karmakar et al. 2019) and viral infection (Xu et al. 2013). Therefore, photosynthetic activity varies depending on the feeding style of the pathogen, which is consistent with the response in other species (for review Chen et al. 2019). In addition, the appearance of cell death lesions during the necrotrophic infection stage is likely to underly the significantly lower level of photosynthesis-related enzymes and metabolites.

ROS exert a positive effect on defense to various pathogens by acting as signaling molecules or inhibiting pathogens by inducing local cell death. However, enhanced accumulation of ROS might result in cell death, thus facilitating the virus (Xu et al. 2013) and necrotrophic pathogen (Suharti et al. 2016b), or it might suppress normal plant metabolism due to the occurrence of oxidative stress. Therefore, the balance of ROS production and scavenging must be tightly regulated. The global profiling studies reviewed herein support the correlation between ROS and photorespiration and photosynthesis. Particularly, enhanced photorespiration is important for the induction of ROS (Kangasjärvi et al. 2012), whereas ROS, such as H_2_O_2_, suppress photosynthesis and plant processes in response to stress (Xu et al. 2013). ROS are also induced by SA (Li et al. 2012b) or the inactivation of GABA shunt (Suharti et al. 2016b). Although ROS accumulation has been reported in response to most biotic stresses, the timing and intensity of ROS vary significantly depending on the pathogen and cultivar, which present different levels of susceptibility.

Phytohormones are also a key response exploited by rice and pathogens. In order to induce a defense response, rice plants upregulate SA signaling when exposed to *M. oryzae* (Meng et al. 2019; Azizi et al. 2019), JA when exposed to insects (Zhang et al. 2019; Wang et al. 2020), and both SA and JA when exposed to *R. solani* (Ma et al. 2020a). PGPR also support rice defense via the induction of SA, as reported for *Pseudomonas* (Kandaswamy et al. 2019), or the induction of ABA signaling and suppression of SA signaling via the bioactive SM cytosinpeptidemycin in response to *Streptomyces* (Yu et al. 2018). Conversely, ABA and cytokinins are activated during viral infection or during the early stage of *M. oryzae* invasion (Cao et al. 2016) in order to facilitate infection. Viral infection downregulates IAA, GA, JA, and SA (Huang et al. 2018). SA activates ROS faster in resistant cultivars and alleviates the decrease in plant photosynthesis (Li et al. 2012b), whereas JA suppresses photosynthesis (Rakwal and Komatsu 2000). Moreover, ABA signaling influences the calcium (Ca^2+^) signaling that is considered as a front line of signaling events and is involved in resistance response of rice against *M. oryzae* (Wang et al. 2019). In a proteomics study of resistant Gangyuan8 (GY8) and susceptible Lijiangxintuanheigu (LTH) cultivars infected by *M. oryzae*, Ca^2+^ − dependent protein kinase, Ca^2+^ sensor calmodulin, and calmodulin-like protein were particularly found upregulated in LTH but remained the same in GY8, suggesting that the proteins possibly regulate blast resistance negatively in those cultivars (Ma et al. 2020b). Another proteomics study found several Ca^2+^-binding proteins in saliva of phloem-feeding insects and honeydews of BPH and green rice leafhopper, supporting the function of Ca^2+^-binding proteins in counteracting the sieve-tube occlusion defenses in host plants (Will et al. 2013; Zhu et al. 2020). These findings suggest that phytohormone regulation, as part of plant defense mechanisms, via different target pathways is complicated, and that cooperation occurs between the pathways.

Signaling components and SMs are highly diversified molecules, dependent on the type of biotic stress. For example, different interactions between rice and PGPR result in different metabolic changes (Chamam et al. 2013). This is explained by the diverse types and functions of these molecules, especially SMs (Erb and Kliebenstein 2020). SMs are less well-conserved, multifunctional metabolites, which guarantee the response to various biotic factors, but resist manipulation and save costs associated with biosynthesis. Therefore, differences in SMs are associated with the resistance of different cultivars to pathogens at different stages of infection. Moreover, each cultivar has a set of differentially expressed proteins (Prathi et al. 2018), diversifying the response.

### Limitations in Global Proteomics and Metabolomics Studies in Rice

Studying proteins and metabolites is more difficult than studying transcriptomics for several reasons: (1) the complexity of proteins and metabolites with different properties makes them difficult to identify using the same method. In addition, proteins undergo various post-translational modifications, resulting in the generation of different isoforms (for review, see Wu et al. 2016; Tan et al. 2017). (2) Technical obstacles, including an appropriate extraction method, the sensitivity of detection, and the detection of post-translational modifications, can limit the detection of proteins or metabolites present at a low abundance (for review, see Castro-moretti et al. 2020). Due to the cost of producing SMs, rice plants are required to maintain a high level of regulation, processing, and storage, to ensure that some SMs are produced at trace amounts (for review, see Erb and Kliebenstein 2020). To detect those SMs, an appropriate experimental design, pipelines, and standard methods are critical (for review, see Alexander and Cilia 2016; Chen et al. 2019). (3) Lack of an information library or database to identify new molecules results in a comparatively large amount of unknown proteins or metabolites in each study (for review, see Chen et al. 2019; Castro-moretti et al. 2020). For example, 15 out of 21 general differentially expressed proteins were unknown in the study of Zhang (Zhang et al. 2019). Thirty-three identified metabolites were undefined in the study of Madhavan (Madhavan et al. 2019). Additionally, 7% and 9% of metabolites extracted from *R. solani* infection in susceptible and resistant cultivars, respectively, were undefined (Suharti et al. 2016b). Rice plants possess specific metabolites (for review, see Okazaki and Saito 2016), which cannot be identified based on the libraries of other species. However, with recently developed methods, we have made progress and expanded our knowledge in this area, exemplified by the identification of new biomarkers (Agarrwal et al. 2014; Duan et al. 2020)**.**

### Consistency in Global Studies of Transcripts and Proteins

Global studies of proteins and metabolites are usually combined with transcription profiling (Table 1 and Table 2). In general, the fluctuation of major molecules is well corelated with gene expression (Sana et al. 2010; Zha and You 2020); however, inconsistencies have also been observed. Peroxidase expression is not associated with the activity between Nipponbare and *O. meyeriana* (Chen et al. 2016). In the study of Zhang, the expression patterns of four out of eight genes were in contrast to the expression pattens of the proteins (Zhang et al. 2019). A low correlation between mRNA and protein levels has also been observed in half of all genes examined in secreted proteins from rice suspensions (Dong et al. 2017). This may be due to post-transcriptional regulation, for example by RNA binding proteins (Xu et al. 2013), or post-translational modifications, which were recently shown to be a significant response to insect invasion (Zha and You 2020). Furthermore, the challenges of methods used in proteomics have limited the identification of all possible isoforms, consequently influencing correlation studies.

### Use of Proteomics and Metabolomics to Improve Rice Performance

Time-series studies have complemented our understanding on the conflict between pathogens and rice at each stage of infection in susceptible and resistant cultivars. Accordingly, the outcome of this conflict is determined by the up or downregulation of certain molecules as well as the intensity of these molecules. For example, defense-related metabolites, cyanoamino acids, and lipid metabolism were increased in both susceptible and resistant cultivars but were more stable in rice resistant to BPH (Kang et al. 2019). Resistant rice infected with *M. oryzae* displayed a higher sensitivity to SA (Li et al. 2012b), and resistance to *R. solani* is dependent on the stability of JA and lignification (Suharti et al. 2016c). These studies confirmed a potential metabolic target but emphasized the limitations associated with studying and utilizing metabolites, especially phytohormones, whose balance is critical for plant growth and development (Peleg and Blumwald 2011). One necessary approach for more effective outcome from rice-pathogen interaction studies would be integration of omics approaches, such as combining transcriptomics with proteomics or metabolomics (Prathi et al. 2018; Wang et al. 2020) or combining proteomics with metabolomics (Karmakar et al. 2019). Moreover, combining separate studies on specific stressor may serve as excellent approach, even though this may bring out some inconsistency. Resultant proteins and metabolites in response to a common biotic stressor may belong to similar pathways, which will eventually increase the efficacy of outcome to get more detailed insight into the intricate cellular activities during rice responses to that stressor. For instance, two separate proteomics and metabolomics studies on rice response to leafroller insect (*Cnaphalocrocis medinalis*) suggest that the JA biosynthesis pathway related proteins and metabolites are critical for resistance (Cheah et al. 2020; Wang et al. 2020).

The limited identification of molecules in proteomics and metabolomics studies as compared to transcriptomics studies has implied the simpler outcome at the final products than the gene regulation. In this context, metabolomics studies on resistance genes have recalled a long-standing question: whether different genes associated with resistance result in different outcomes via different metabolic pathways. Studies on *Pizt*- and *Pi21*-mediated resistance and basal resistance in dsOW62/76 have suggested a different outcome. If this is the case, SMs represent a tool that is guaranteed to perform well. However, the greatest obstacle is the cost of SM biosynthesis. In most studies, the number of upregulated SMs is greater than the number of downregulated SMs, affirming the tight regulation of SMs due to their cost. Therefore, more studies on resistance genes and how to deploy SMs in plant resistance are needed.

**Fig. 1:**
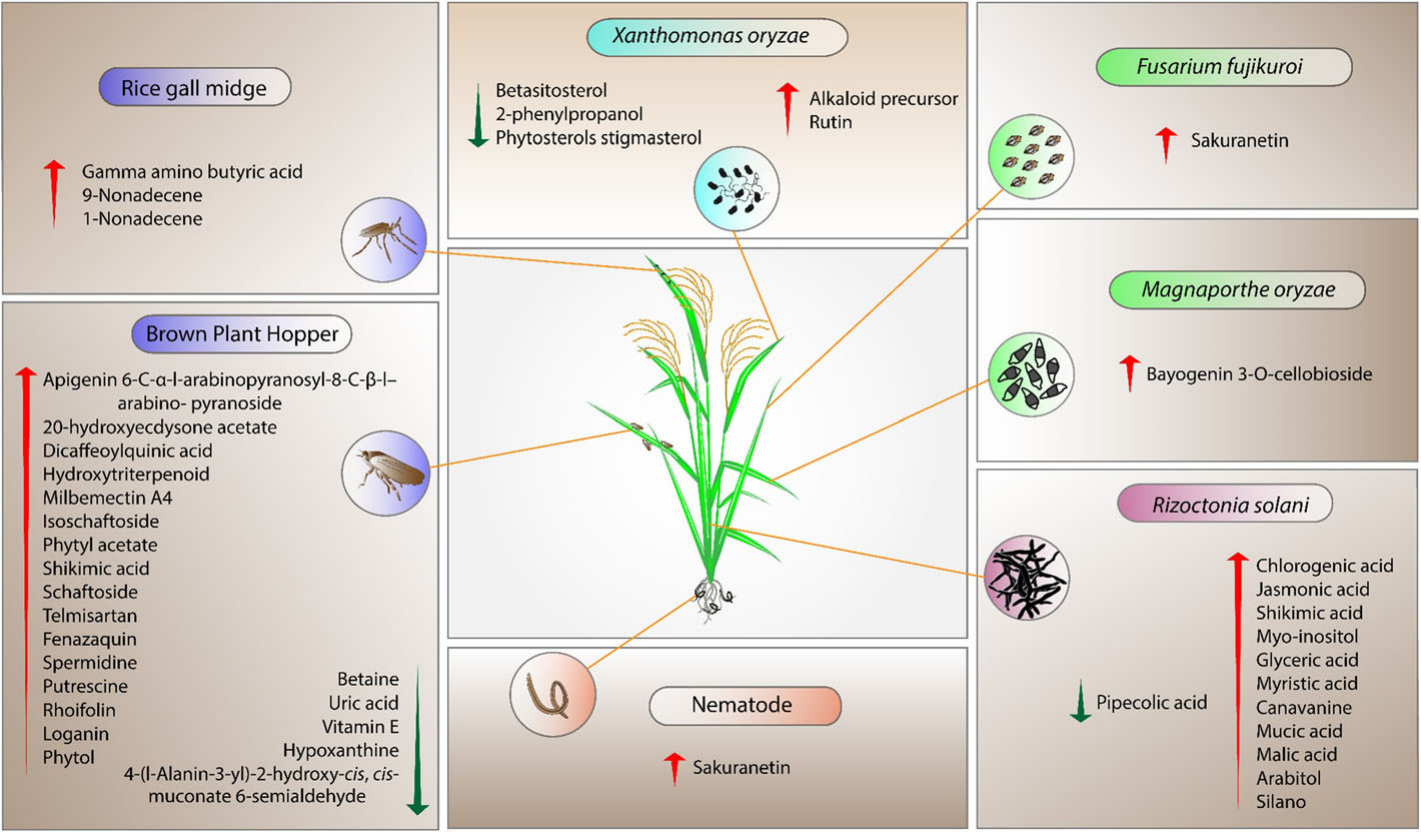
Potent metabolites linked to stress resistance in rice. Those metabolites were specifically increased (red arrow) or decreased (green arrow) in resistant plants as compared to susceptible plants in indicated studies (Table 2, marked by ^a^). SA: salicylic acid, JA: jasmonic acid

The application of metabolomics to improve plant performance to stresses has been previously suggested (Hong et al. 2016). An example was proposed by Kushalappa and Gunnaiah (2013), who suggested 10 heuristic steps to streamline metabolomics-proteomics studies to identify resistance genes. The main difference in the metabolic profiles of resistant and susceptible cultivars provided us with scaffolds to produce stable resistant rice, which is sometimes confined by a resistance gene approach. Thus, we collected the potent metabolites responsible for resistance to different stressors, as illustrated in Fig. 1. These metabolites are specifically induced or reduced in resistant rice cultivars (Table 2). Due to the multiple functions of primary metabolites in rice, we limited our analysis to SMs or primary metabolites that function in resistance via a non-primary pathway. These SMs provide targets for further investigation and use.

## Abbreviations

2-DE: Two-dimensional gel electrophoresis
ABA: Abscisic acid
ETI: Effector-triggered immunity
GC: Gas chromatography
iTRAQ: Isobaric tags relative and absolute quantification
JA: Jasmonic acid
LC: Liquid chromatography
MS: Mass spectrometry
PAMP: Pathogen-associated molecular pattern
PTI: PAMP-triggered immunity
SA: Salicylic acid
SM: Secondary metabolite
TF: transcription factor
